# Testing gene-environment interactions in gene-based association studies

**DOI:** 10.1186/1753-6561-5-S9-S26

**Published:** 2011-11-29

**Authors:** Xuefeng Wang, Huaizhen Qin, Nathan J Morris, Xiaofeng Zhu, Robert C Elston

**Affiliations:** 1 Department of Epidemiology and Biostatistics, Case Western Reserve University, 2103 Cornell Road, Cleveland, OH 44106-7281, USA

## Abstract

Gene-based and single-nucleotide polymorphism (SNP) set association studies provide an important complement to SNP analysis. Kernel-based nonparametric regression has recently emerged as a powerful and flexible tool for this purpose. Our goal is to explore whether this approach can be extended to incorporate and test for interaction effects, especially for genes containing rare variant SNPs. Here, we construct nonparametric regression models that can be used to include a gene-environment interaction effect under the framework of the least-squares kernel machine and examine the performance of the proposed method on the Genetic Analysis Workshop 17 unrelated individuals data set. Two hundred simulated replicates were used to explore the power for detecting interaction. We demonstrate through a genome scan of the quantitative phenotype Q1 that the simulated gene-environment interaction effect in the data can be detected with reasonable power by using the least-squares kernel machine method.

## Background

There is continuing interest in the investigation of interactions in human genetics, including gene-environment and gene-gene interactions, on the assumption that they play an important role in understanding complex traits. Considerable challenges still exist, however, from the definition of statistical interaction to its analysis and interpretation [1]. As defined by statisticians, interaction is traditionally a departure from additivity incorporated into a linear regression model (logistic regression for binary traits) as one or more product terms. For example, we may model:(1)

where *y_i_* is the quantitative trait outcome of the *i*th individual, *x_ji_* are binary indicator variables of genotypes or exposures, *β*_1_ and *β*_2_ are regression coefficients of the main effects of genotypes or exposures, and *β*_3_ is an interaction effect term. In genetic association studies, we usually wish to achieve two purposes by incorporating such an interaction term: first, improving the power to detect a causal gene with interaction effects; and, second, detecting an interaction effect per se, which hopefully will allow us to elucidate biological interaction. Testing for the first purpose (i.e., testing for association with genotypes at a locus while allowing for an interaction effect, either with genotypes at another locus or with an exposure) corresponds to the test *H*_0_: *β*_1_ = *β*_3_ = 0 or *H*_0_: *β*_2_ = *β*_3_ = 0 (with two degrees of freedom), whereas testing for the second purpose corresponds to testing whether *β*_3_ = 0 (with one degree of freedom). It is our purpose here to investigate whether similar procedures can be applied in the setting of nonparametic regression. Given the complex nature of interaction effects, it may be necessary to consider a more flexible parameterization of statistical interaction (which nonparametric regression allows) than just the product of first-order terms.

Our analysis is also motivated by gene-based association studies. Like the Genetic Analysis Workshop 17 (GAW17) data, many current studies provide both single-nucleotide polymorphisms (SNPs) and their affiliated gene information. Gene-centric tests that consider association between a trait and all markers within a gene region have become an important complement to traditional single-locus tests. Chatterjee et al. [2] proposed a logistic regression model that includes all pairwise interactions between SNPs across two genes or between all SNPs in one gene and an environment exposure. The estimation and inference were made feasible by using Tukey’s parsimonious one-degree-of-freedom model of interaction. Two inherent limitations of using Tukey’s model are (1) that nonremovable interactions and interactions involving factors with small marginal effects are not detected and (2) that the method may be more suitable for a candidate gene study, given that the evaluation of the test statistic is computationally demanding because the standard score test is not applicable. To allow the investigation of more interaction models, we propose a different solution that is computationally attractive and based on a least-squares kernel machine (LSKM).

The kernel machine (such as the well-known support vector machine) originated from machine learning techniques and has attracted considerable interest in recent years. It is being increasingly applied to genetics. The key idea behind kernel machines is to implicitly transform the original input data to a higher-dimension nonlinear space that allows a more efficient exploration of data patterns for classification and model fitting. Nonparametric regression implemented by an LSKM has also been proposed as a promising tool in SNP-set gene- and pathway-based association studies [3-5]. An LSKM-based regression can test for the overall association of a gene to a disease by using genetic information from multiple SNPs simultaneously, thus providing a test statistic with an adaptively estimated number of degrees of freedom. By specifying a flexible kernel function, this method also allows for modeling interaction effects in many forms other than the product form. In this report we focus on the analysis of quantitative phenotype Q1 in the GAW17 data set with an LSKM-based method that shows the greatest promise.

## Methods

We use a notation similar to that of Kwee et al. [4]: Suppose that there are *p* SNPs within a gene; *g_i,k_* is the genotype of individual *i* at SNP *k* (coded 0, 1, or 2, reflecting the number of copies of the minor allele), **g***_i_* = (*g_i_*_,1_, *g_i_*_,2_, …, *g_i,p_*) is a *p* × 1 vector of genotypes of the SNPs in the gene for individual *i*, and *X_i_* is a *q* × 1 vector of covariates (including Sex, Age, Smoking, and principal components to allow for population stratification). The basic semiparametric regression model for the outcome of an individual can then be written:(2)

where **β** is a *q* × 1 vector of covariate coefficients and *h*(·) is a nonparametric smoothing function that allows a flexible modeling of the influence of the genotype information **g***_i_* on the trait value or disease risk (for which the outcome is replaced by logit[*P*(*y_i_* = 1)]). Our primary interest is to test whether the overall effect of a gene or SNP set is 0, that is, whether *h*(**g***_i_*) = 0.

Under the LSKM framework, the function *h*(·) can be expressed as a linear combination of kernels:(3)

for some *α_i_*, …, *α_n_* (see Liu et al. [3] for mathematical details). The choice of kernel function determines the type and complexity level of the relationship between the genotypes and the trait. The two kernel functions used most often are the (*d*th) polynomial kernel:(4)

and the Gaussian kernel:(5)

for individuals *i* and *i*′. For a quadratic kernel (*d* = 2), assuming **g***_i_* = (*g_i_*_,1_, *g_i_*_,2_) , it is easy to show that:(6)

where the function *φ*(**g**) projects the data (*g_i_*_,1_,*g_i_*_,2_)*^T^* to . Therefore kernel functions can implicitly map input data to a higher-dimension inner product space (kernel trick).

Intuitively, a kernel function can also be used as a similarity measure between two individuals. For example, the linear kernel function  can be shown to be analogous to a covariance when **g** is centered. Based on this idea, a kernel function can be constructed using the identify-in-state (IIS) sharing information across the region:(7)

where:(8)

[5]. Liu et al. [3] showed that this estimation and inference can be done analogously in the framework of a linear mixed model, which is much easier to implement. By treating the nonparametric function *h*(·) as a subject-specific random effect, Eq. (2) can be rewritten for all *n* observations as:(9)

where *X* is the matrix of covariates, **h** is a vector of random effects resulting from all SNPs in the region, following a distribution with mean 0 and variance *τ***K**, and **e** ~ *N*(0, σ^2^**I**). It has been shown that the best linear unbiased estimates of the fixed effects **β** and random effects **h** under restricted maximum likelihood (REML) share a common mathematical form with the LSKM estimates. It follows that the test of *H*_0_: *h* = 0 is equivalent to testing *H*_0_: *τ* = 0. A score statistic for this purpose is given by , which is distributed as a sum of weighted chi-square variables and can be approximated by a scaled chi-square distribution using Satterthwaite’s procedure [4,6] through matching the first two moments. These steps share many features with variance component methods [7].

To evaluate the performance of LSKM methods to incorporate and detect gene-environment interactions, we use three sets of models in analyzing the gene-based SNP sets in the GAW17 data. Each model is tested using linear and quadratic kernel functions. First, the baseline model (Eq. (2)) is considered without including any gene-environment interaction effects. These results are used mainly for comparison with interaction models, but they can also help test the efficacy of the nonparametric methods to detect a gene’s main effect. Furthermore, to address the rare variant issue in the GAW17 data set, we further introduce a combined genotype *c_i_*, which is the sum of the elements in **g***_i_* for one gene. The corresponding semiparametric regression model is then:(10)

Second, to detect gene-smoking interaction per se, as discussed in the introduction, we formulate the following two testing models in a similar way but using parametric regression:(11)(12)

where **γ** is the vector or scalar regression coefficient measuring respectively the main effects of **g***_i_* or *c_i_*, respectively, and **t***_i_* is composed of the product term(s) between smoking status and genotypes **g***_i_* or smoking status and genotype sum *c_i_*. The main effect of smoking is included in the fixed effect vector **β** in models (11) and (12).

Finally, we consider two other models (Eqs. (13) and (14)) for a joint test of marginal and interaction effects, in which genotypes (**g***_i_* or *c_i_*), smoking status (*s_i_*), and the interaction term (**t***_i_*) are all put into the function *h*(·), for which we use a quadratic kernel function:(13)(14)

where(15)

and(16)

## Results

In the initial stage of our analysis, we tested three kernel functions on a subset of genes (one gene at a time) and found that the quadratic and Gaussian kernels produced consistent results but that the quadratic kernel was computationally much faster. Therefore, using a quadratic kernel, we performed a genome-wide scan using each of the 200 simulated replicates. Note that there was no need to put the product terms into the nonparametric function in models (13) and (14) when the Gaussian kernel was used because the Gaussian kernel automatically allows searching through a more inclusive space. Through this analysis, we answer the two separate questions asked in the introduction: (1) What is the power of the LSKM-based method to detect a gene-environment interaction effect per se, based on models (11) and (12); and (2) does incorporating interaction terms into the LSKM improve the power of detecting a true gene with interaction effects, based on models (13) and (14)?

**Figure 1:**
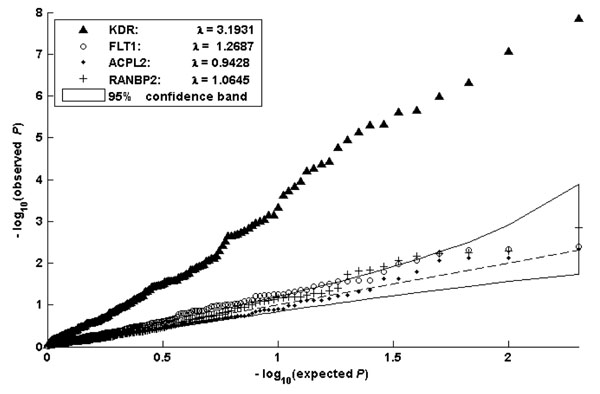
**Testing interaction between smoking and genes.** The Q-Q plots show the distribution of –log_10_*p*-values from 200 replicates based on model (12) for four genes: *KDR* (true interaction gene), *FLT1* (large marginal effect gene), and two noise genes. The dashed line is a reference line with slope 1, and the solid line region corresponds to the 95% confidence band obtained under the null hypothesis (no interaction).

Here, we chiefly report the results for the gene *KDR* and a few other genes acting on the quantitative trait Q1, because analysis of most of the other genes and traits showed no signals in terms of detecting interaction effects. In general, the models based on the genotype sum (models (12) and (14)), yielded greater power than the models using raw genotype scores. Figure 1 shows the quantile-quantile (Q-Q) plot of the distribution of –log_10_*p*-values, based on model (12), for the genes *KDR*, *FLT1* (a gene with a large marginal effect), and two noise genes. The curve for gene *KDR* is clearly separated from the other three curves, indicating that this model has substantial power to reject the null hypothesis *h*(*t_i_*) = 0. Most points along the curve for *FLT1* lie in or near the 95% confidence band but above those of two other genes, suggesting a slightly inflated type I error.

Similarly, we can explore the improvement in power of a joint test (models (13) and (14)) versus a main effect model (model (10)) by comparing the resulting two curves in Q-Q plots. We found that both curves for the *KDR* gene lay above the 95% confidence band and were visually separated. The same pattern as that found for the other genes was found without incorporating interaction effects, for example, *FLT1*. Therefore the deviation of these curves cannot be directly attributed to an increase in power.

**Figure 2:**
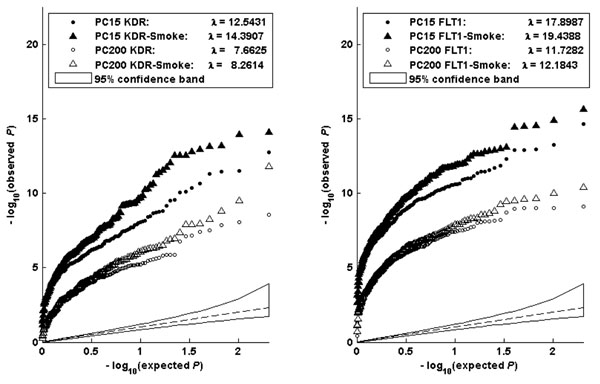
**Joint testing of gene-environment interaction and main effects**. The Q-Q plots compare the distribution of association –log_10_*p*-values for gene *KDR* (left panel) and *FLT1* (right panel) derived from model (10) (circles) and model (14) (triangles) adjusted for 15 principal components (solid triangles and circles) or 200 principal components (open triangles and circles).

We further examined these models by adjusting for population structure. Figure 2 shows the Q-Q plots of the association –log_10_*p*-values for the genes *KDR* (left panel) and *FLT1* (right panel) after including the first 15 principal components (PCs) to allow for population stratification (solid points) and 200 components (open points). Each plot thus contains two sets of curves, in which the results of model (10) and model (14) are represented by circles and triangles, respectively. We can see that, by increasing the number of PCs, the difference between the curves becomes smaller, but the difference for gene *FLT1* tends to shrink faster than that for gene *KDR*. However, even with 200 PCs adjusted in gene *FLT1*, the curve of model (14) is still above that of model (10).

## Discussion

The study of interaction in human genetic association studies faces many challenges that are well known in the field, such as issues of computational burden, model dependency, and multiple testing [8-10]. A few additional issues arise in the analysis of gene-environment interaction using the GAW17 simulated data. First, as a major theme of GAW17, a large proportion of rare variant SNPs are contained in the data. This considerably reduces the power of SNP-based association tests that test only main effects—not to mention the interaction, which suffers more from a sparsity issue. A simple but practical solution is to combine genotypes within a gene, as we demonstrated in our analysis. Other genotype collapsing or aggregating methods, such as adaptive and weighted-sum methods, may also be applied. The analysis of interaction has been largely restricted by the simulation scheme used in generating the GAW17 data: Only one gene is simulated with a gene-environment interaction. The GAW17 data thus do not enable a systematic comparison of different methods or models. The confounding factor of population structure (present though not planned) has further complicated the analysis and interpretation of our results. Depending on the interaction model, any hidden population structure may yield false-positive results in a joint analysis of main and interaction effects, as shown in our results.

Despite all these restrictions, through our analysis we have demonstrated the advantages of the LSKM-based method. First, the method provides a flexible modeling and testing framework for multilocus and gene-based association studies, which allows the analysis of both quantitative and binary traits and the easy incorporation of covariates; the method can automatically reduce the degrees of freedom of the test by properly accounting for the correlation structure among markers. Second, various interaction models and nonlinear effects can be implicitly defined by specifying different kernel functions. Third, the score-based statistic makes the method’s implementation computationally efficient and thus suitable for both candidate genes and a genome-wide scan. The procedure described in this paper can be readily applied to gene-gene interaction. More simulation scenarios will be required in a future study to explore the performance of different gene collapsing methods and kernels. For example, a weighted version of the IIS kernel can be considered to emphasize the similarity between rare-variant SNPs [4,5]. One possible extension would be to include a polygenic control term in the model (similar to a variance component method) so that information from family and unrelated case-control data can be combined. It would also be of interest to test whether the LSKM-based interaction model can be adapted for use in other classes of genomic similarity methods [11,12].

## Conclusions

By incorporating interaction terms, explicitly or implicitly, and using LSKM-based regression methods, we were able to detect signals for the interaction effects simulated in forming the quantitative trait. We were able to gain some power by jointly testing the main effects and interactions, but the results were confounded by the population structure that exists in the GAW17 data.

## Competing interests

The authors declare that they have no competing interests.

## Authors’ contributions

XW and HQ participated equally in the conception and design of the study, and carried out the statistical analyses. XW and RCE drafted the manuscript. NJM participated in the design of the study and editing the manuscript. XZ participated in the design of the study. RCE did the final editing of the manuscript. All authors read and approved the final manuscript.
